# The effect of mental fatigue on critical power during cycling exercise

**DOI:** 10.1007/s00421-017-3747-1

**Published:** 2017-11-09

**Authors:** Hawbeer Salam, Samuele M. Marcora, James G. Hopker

**Affiliations:** Endurance Research Group, School of Sport and Exercise Sciences, University of Kent, Chatham Maritime, Kent, UK

**Keywords:** RPE, Time-to-exhaustion, Maximal exercise, Perception of effort, Endurance

## Abstract

**Purpose:**

Time to exhaustion (TTE) tests used in the determination of critical power (CP) and curvature constant (W′) of the power–duration relationship are strongly influenced by the perception of effort (PE). This study aimed to investigate whether manipulation of the PE alters the CP and W′.

**Methods:**

Eleven trained cyclists completed a series of TTE tests to establish CP and W′ under two conditions, following a mentally fatiguing (MF), or a control (CON) task. Both cognitive tasks lasted 30 min followed by a TTE test. Ratings of PE and heart rate (HR) were measured during each TTE. Blood lactate was taken pre and post each TTE test. Ratings of perceived mental and physical fatigue were taken pre- and post-cognitive task, and following each TTE test.

**Results:**

Perceived MF significantly increased as a result of the MF task compared to baseline and the CON task (*P* < 0.05), without a change in perceived physical fatigue (*P* > 0.05). PE was significantly higher during TTE in the MF condition (*P* < 0.05). Pre-post blood lactate accumulation was significantly lower after each TTE in MF condition (*P* < 0.05). HR was not significant different between conditions (*P* > 0.05). Neither cognitive task induced any change in CP (MF 253 ± 51 vs. CON 247 ± 58W; *P* > 0.05), although W′ was significantly reduced in the MF condition (MF 22.8 ± 4.5 vs. CON 29.3 ± 6.3 kJ; *P* < 0.01).

**Conclusion:**

MF has no effect of CP, but reduces the W′ in trained cyclists. Lower lactate accumulation during TTE tests following MF suggests that cyclists were not able to fully expend W′ even though they exercised to volitional exhaustion.

## Introduction

The critical power (CP) concept is based upon a mathematical model of the hyperbolic relationship between work done and time to exhaustion (TTE). Since the early work of Monod and Scherer (1965), it has been accepted that the hyperbolic relationship is reflective of physiological responses to endurance exercise, where CP represents the highest sustainable rate of aerobic metabolism (Gaesser and Wilson 1988; Hill 1993). Indeed, Poole et al. (1988) demonstrated that if endurance exercise is performed at the CP, following the initial “fundamental” increase in oxygen consumption ($\dot {V}{{\text{O}}_{\text{2}}},$) a delayed steady state is achieved within a few minutes. Moreover, the increase in $\dot {V}{{\text{O}}_{\text{2}}}$ is replicated by the blood lactate response, which following an initial rise at the start of endurance exercise, stabilizes after some minutes. When endurance exercise was performed 5% above the CP, $\dot {V}{{\text{O}}_{\text{2}}}$ continued to increase until $\dot {V}{{\text{O}}_{\text{2}}}$ was reached, and blood lactate continued to rise until the participants were no longer able to exercise. Thus within the hyperbolic work-time relationship, the asymptote is defined as the CP. The curvature constant of the model is known as the W′ and represents the amount of work that can be performed above the CP, regardless of the chosen work rate above CP (Moritani et al. 1981; Monod and Scherrer 1965; Poole et al. 1988). W′ has been attributed to the depletion of intramuscular phosphocreatine (PCr) and glycogen with accumulation of fatigue-related muscle metabolites including hydrogen ion (H^+^) and inorganic phosphate (P_i_) (Fitts 1994).

The work–time relationship does not directly measure the physiological parameters that are purported to contribute to its determination. Instead, endurance performance, often measured as TTE, is plotted against the externally measured work done (per unit time) to determine the CP and W′. However, TTE tests are strongly influenced by perception of effort, defined as “the conscious sensation of how hard, heavy and strenuous exercise is” (Marcora 2009a). The psychobiological model of endurance performance has been shown to provide a valid explanation of the effects of both psychological (Marcora et al. 2009b; Pageaux et al. 2013) and physiological (Marcora et al. 2008) manipulations on endurance performance during TTE tests. The psychobiological model (Marcora 2009a) is an effort-based decision-making model based on motivational intensity theory (Brehm and Self 1989), and postulates that the point of exhaustion during a TTE test is not caused by muscle fatigue, rather the conscious decision to disengage from the endurance task. In highly motivated individuals, this effort-based decision is taken when their perception of effort is maximal and continuation of the endurance task seems impossible. Marcora et al. (2009b) conducted the first experimental study on the effect of prolonged mental exertion on endurance performance. The investigators induced mental fatigue in a group of healthy but untrained participants using a prolonged and demanding cognitive task and found a significant reduction in TTE during subsequent high-intensity cycling exercise. Interestingly, the reduction in TTE was independent of alterations in cardiorespiratory or metabolic responses to high-intensity cycling exercise, instead being explained by a higher perception of effort experienced by mentally fatigued participants. More recent studies have also demonstrated a negative effect of prolonged mental exertion on self-paced endurance tasks (Pageaux et al. 2014; Smith et al. 2015). Using a randomized cross-over design, Brownsberger et al. (2013) demonstrated a reduced self-paced cycling exercise performance following a cognitively demanding task, compared to a control condition. Specifically, following prolonged mental exertion, participants had higher self-reported sensations of fatigue, and produced less physical work during two bouts of self-paced cycling exercise equivalent to a rating of perceived exertion (RPE) of 11 and 15 respectively.

Therefore, it is plausible to speculate that a higher perception of effort may limit TTE and subsequently alter the CP and W′, independently of changes within the aforementioned underlying muscle physiology. Indeed, Nakamura et al. (2005) have previously demonstrated strong correlations between perception of effort and critical power determined from repeated TTE testing. As a consequence, we hypothesised that mental fatigue from a prolonged and demanding cognitive task would reduce subsequent TTE during cycling exercise, and therefore alter the CP and W′.

## Methods

### Participants

Eleven well-trained male cyclists (age 38 ± 6 years; body mass 76.5 ± 9.6 kg; $\dot {V}{{\text{O}}_{{\text{2peak}}}}$ 60.5 ± 4.1 ml kg^−1^ min^−1^) who completed more than 5 h of training per week, for at least 3 years were recruited to take part in the study.

### Ethical approval

Following institutional ethical approval in line with the Declaration of Helsinki, participants provided written informed consent to participate. All participants were given written instructions describing the procedures related to the study but were naive of its aims and hypotheses. Participants were told that the study was investigating the effect of two different cognitive tasks (computerized task vs. reading magazines) on the physiological responses to endurance cycling exercise. At the end of the last visit participants were debriefed as to the true nature of the study.

### Study protocol

**Fig. 1:**
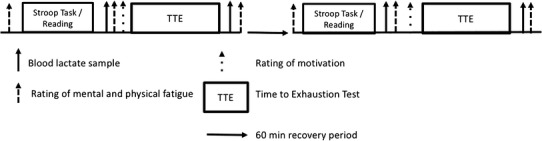
Overview of the study protocol

Each participant visited the exercise testing laboratory on five separate occasions with minimum of 2 days between visits. At the first visit, participants completed an incremental exercise test to identify $\dot {V}{{\text{O}}_{{\text{2peak}}}}$ (see “[Sec Sec6]”) and were familiarized with the time to exhaustion tests to be used at subsequent visits. On arrival at the laboratory, participants were asked to provide a capillary blood sample for the determination of resting blood lactate and glucose concentration (Biosen C-Line; EKF Industrie, Electronik GmbH, Barleben, Germany). At this time, a heart rate monitor (S810i, Polar, Kempele, Finland) was fitted to the participant to record heart rate (HR) continuously throughout the visit. During the experimental visits (see Fig. 1), participants completed two randomly assigned and counterbalanced TTE tests. The TTE tests were performed following either a 30 min cognitively demanding computerized task designed to elicit mental fatigue, or a control task involving 30 min of reading a magazine (see “Experimental conditions”). After the first TTE, participants had a 30 min period of passive recovery, before undertaking a further 30 min of the same experimental manipulation prior to completing the second TTE test. TTE tests were conducted at power outputs equivalent to 40, 60, 80% of the difference between the gaseous exchange threshold (GET) and $\dot {V}{{\text{O}}_{{\text{2peak}}}}$, as well as at the work rate equivalent to $\dot {V}{{\text{O}}_{{\text{2peak}}}}$ (see “TTE testing”). Power outputs (40%Δ 60%Δ, 80%Δ or 100% $\dot {V}{{\text{O}}_{{\text{2peak}}}}$) were randomized and divided into two visits per condition (CON and MF). Participants were asked to rate their perceived mental and physical fatigue prior to, and following each experimental manipulation, and after each TTE test. They were also asked to rate their level of motivation immediately prior to each TTE test (see “Psychological measurements”). A capillary blood sample was also taken prior to and following each TTE test.

### $\dot {V}{{\text{O}}_{{\text{2peak}}}}$ determination

Upon arriving at the laboratory, participant’s height and weight (Seca, Hamburg, Germany) were measured. Following a 5-min warm-up at 100 W, participants completed an incremental exercise test until volitional exhaustion using a 25 W per minute ramp rate. Expired gases were assessed on a breath-by-breath basis using an online gas analyser (Metalyzer, Cortex Biophysik GmbH, Leipzig, Germany), throughout the test. Participants cycled on an electromagnetically braked cycle ergometer (Schoberer Rad Messtechnik, Jülich, Germany), with power output being recorded continuously throughout the test. Prior to the test the ergometer was adjusted for each participant, and settings were recorded to allow reproduction at each subsequent visit. Participants were also given standard instructions for the overall RPE using the 15-point Borg scale (Borg 1998). Each participant was then subsequently asked to rate their perceived effort at each minute during the test. HR was monitored continuously throughout the test using a chest strap and watch (S810i, Polar, Kempele, Finland). Throughout the visit laboratory conditions remained stable, and participants were cooled using electric fans. Following the test, $\dot {V}{{\text{O}}_{{\text{2peak}}}}$ was identified as the highest $\dot {V}{{\text{O}}_{\text{2}}}$ attained during a 60 s period in the test. For setting work rates for the subsequent TTE tests, it was necessary to determine the GET using a cluster of measurements, including the first disproportionate increase in CO_2_ production ($\dot {V}{\text{C}}{{\text{O}}_{\text{2}}}$) from visual inspection of individual plots of $\dot {V}{\text{C}}{{\text{O}}_{\text{2}}}$ vs. $\dot {V}{{\text{O}}_{\text{2}}}$, an increase in ventilation (VE) over oxygen consumption (VE/$\dot {V}{{\text{O}}_{\text{2}}}$) with no increase in VE/VCO_2_, and an increase in end-tidal O_2_ tension with no change in end-tidal CO_2_ tension. Subsequently 40%Δ (the work rate at GET plus 40% of the difference between the work rate at the GET and the work rate at $\dot {V}{{\text{O}}_{{\text{2peak}}}}$), 60%Δ, 80%Δ, and the work rate at $\dot {V}{{\text{O}}_{{\text{2peak}}}}$ were calculated.

### TTE testing

Participants were positioned on the same cycle ergometer used for the incremental exercise test and instrumented for the physiological measurements before starting the TTE test, 15 min after the end of experimental manipulation. The constant power cycling TTE test consisted of a 3-min warm-up at 40% of peak power output followed by a rectangular workload corresponding to 40%Δ 60%Δ, 80%Δ or 100% $\dot {V}{{\text{O}}_{{\text{2peak}}}}$. Pedal cadence was freely chosen between 60 and 100 rev min^−1^ and was recorded continuously throughout each test. Time to exhaustion was measured from the start of the rectangular workload until the pedal cadence was less than 60 rev min^−1^ for more than 5 s despite standardized verbal encouragement (Andreacci et al. 2002). Heart rate was recorded continuously throughout all TTE tests, and RPE was taken during the last 15 s of every minute. To account for the different exercise durations, HR and RPE data were subsequently plotted against TTE with the slope of the relationship being calculated. During all exercise tests, participants cycled in an air-conditioned laboratory and were cooled using an electric fan. Linear regression was used to provide estimates of CP and W′ from the four TTE tests using both the work-time [P = W′ +  (CP·t)] model (Hill 1993).

### Experimental conditions

The MF condition comprised of 30 min of engagement with an modified version of the Stroop word-colour task. Participants were seated comfortably in an isolated quiet room while performing the Stroop task on a computer. Four different words (yellow, green, blue, and red) were consecutively showed on the screen until the participant confirmed an answer, and were followed by a 1500 ms interval. Participants were instructed to press one of four coloured buttons on the computer keyboard (yellow, green, blue, and red) with the correct response being the button conforming to the ink colour (either yellow, green, blue, or red) of the word presented on the screen. For example, if the word ‘red’ appeared in green ink, participant should press the green button. If the ink colour was blue, the subject should follow pressing the button linked to the real colour of the word, not the ink meaning (e.g. if the word blue appears in red, the button blue has to be pressed). The computer selects the colours presented in word randomly (100% incongruent). To ensure that the task was understood properly, participants performed 5 min of the task as familiarisation before starting the actual task. Participants were told to answer as accurately and quickly as possible following the presentation of each word. Feedback was presented on the screen after each selection to inform the participants of incorrect or correct answer, accuracy and speed response. E-Prime software (Psychology Software Tools, Pittsburgh, PA, USA) was used to develop the task, and analyse the reaction time and accuracy scores. The HR response was measured throughout the task. During the control condition participants sat quietly and read a fitness magazine for a period of 30 min. During control condition, HR was continuously recorded.

### Psychological measurements

Participants were asked to rate “how do you feel right now?” in terms of both mental and physical fatigue before and after each experimental condition, and after each TTE test. The two items were answered on a 10-point scale (1 = not at all, and 10 = extremely). Participants were also asked to rate their level of motivation in relation to the upcoming TTE test immediately prior to the test. The ten-point scale ranged from 1 = not motivated, to 10 = extremely motivated.

### Statistical analysis

Assumptions of statistical tests such as normal distribution and sphericity of data were checked as appropriate. Two-way repeated-measures ANOVA (2 condition × 4 work rates) was used to assess differences in recorded TTE, blood lactate accumulation, RPE and heart rate slope responses. Differences in perceived mental and physical fatigue, and motivation were also assessed using two-way repeated-measures ANOVA but with the additional component of measurement time point (2 condition × 4 work rate × 3 time point). Greenhouse–Geisser correction to the degrees of freedom was applied when violations to sphericity were present. CP and W′ data between conditions were analysed by paired *t* tests. Statistical analyses were performed using the software program SPSS, version 21.0 (Statistical Package for Social Science, Chicago, Illinois, USA). Statistical significance was accepted at an alpha value of *P* < 0.05. All data are presented as means ± standard deviation (SD) unless stated otherwise.

## Results

### Manipulation check

**Fig. 2:**
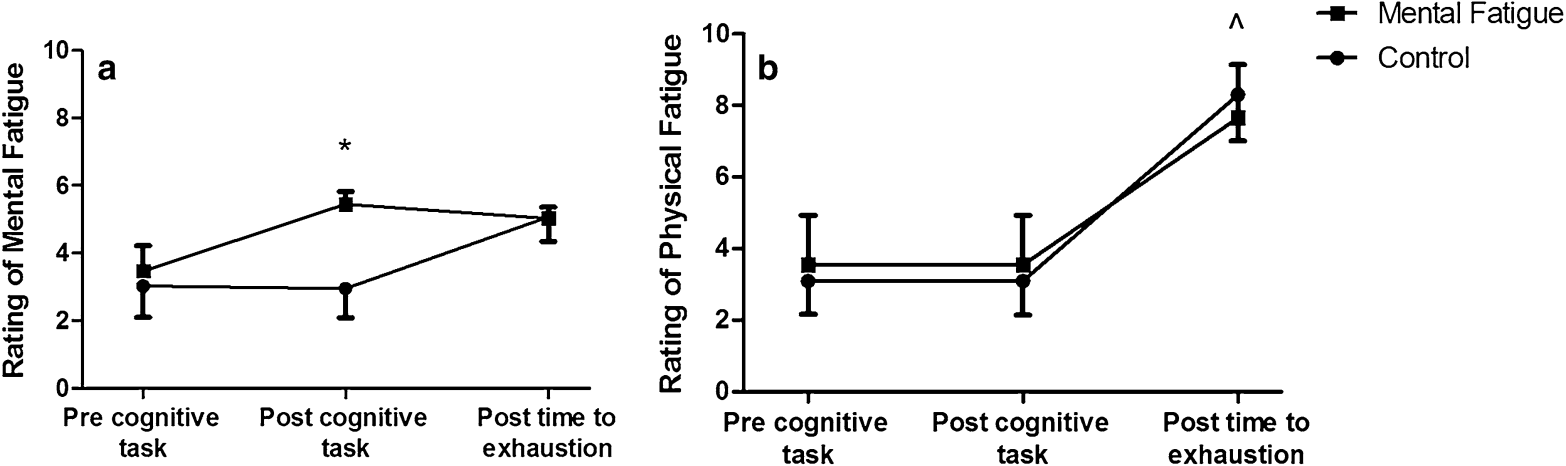
Effects of experimental condition on perception of: **a** perceived mental fatigue; **b** perceived physical fatigue over all tests (40%Δ 60%Δ, 80%Δ and 100% $\dot {V}{{\text{O}}_{{\text{2peak}}}}$). *Significant effect of condition (*P* < 0.05); ^significant effect of time point (*P* < 0.05)

A 30-min Stroop task was used to induce mental fatigue in the participants. There was no condition x intensity x time point, or condition x intensity interaction found for perception of mental fatigue (*P* > 0.05). However, a significant interaction effect was found for condition x time point (*P* < 0.01; Fig. 2a). A significant main effect of condition was found; participants rating of perceived mental fatigue increased after the mentally fatiguing task compared to the CON task (*P* < 0.01). There was no significant condition x intensity x time point, or condition x intensity (*P* > 0.05). However, a significant interaction effect of condition × time point (*P* = 0.04) was found with greater levels of perceived physical fatigue being evident after the TTE tests in both conditions (Fig. 2b).

There was no condition x intensity interaction effect (*P* > 0.05) for level of motivation prior to the TTE tests. Level of motivation was not significantly different following MF or CON conditions immediately prior to the TTE tests. The grand mean for level of motivation was 7.8 ± 0.5 vs. 8.4 ± 0.4 for MF vs. CON, respectively (*P* > 0.05).

### Effects of experimental condition on TTE

Participant maximal power output from the incremental ramp test was 394 ± 47 W, with the resultant TTE power outputs being 289 ± 45, 326 ± 47, 360 ± 46 and 394 ± 47 W 40%Δ 60%Δ, 80%Δ and 100% $\dot {V}{{\text{O}}_{{\text{2peak}}}}$, respectively. There was no interaction effect between condition and intensity (*P* > 0.05), although TTE was significantly reduced following mental fatigue at each exercise intensity compared to CON (*P* < 0.01; Table 1).

**Table 1:** Time to exhaustion (TTE) (s) at 40%Δ 60%Δ, 80%Δ and 100% for the mental fatigue (MF) and control (CON) conditions

TTE (s)	40%Δ	60%Δ	80%Δ	100% $\dot {V}{{\text{O}}_{{\text{2peak}}}}$
CON	720 ± 180	422 ± 88	275 ± 58	190 ± 38
MF	648 ± 171*	341 ± 84*	231 ± 65*	156 ± 38*

*Significant shorter than CON condition (*P* < 0.05)

### Effects of experimental condition on CP and W′

**Fig. 3:**
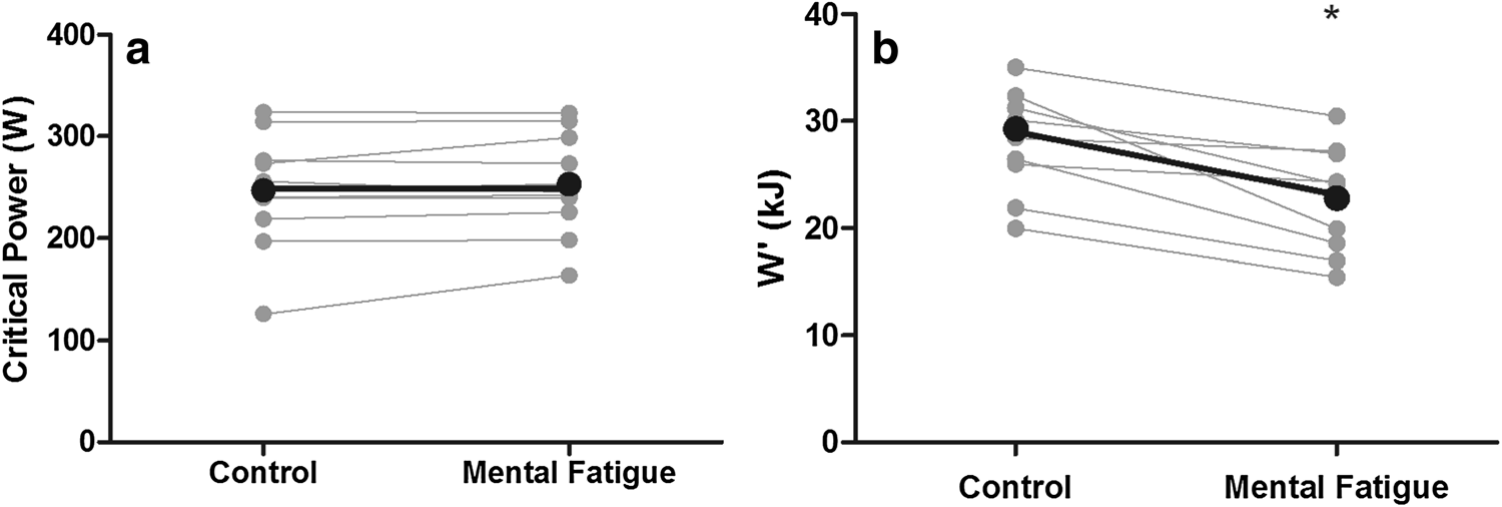
Effects of experimental condition on: (**a**) CP and (**b**) W′ calculated using the work–time model. *Significant effect of condition (*P* < 0.05)

CP was not significantly different between MF and CON conditions (MF 253 ± 51 W vs. CON 247 ± 58 W; *P* > 0.05; see Fig. 3a). However, there was a significant reduction in estimated W′ following MF (MF 22.8 ± 4.5 kJ vs. CON 29.3 ± 6.3 kJ; *P* < 0.01; see Fig. 3b), with all participants demonstrating a lower curvature constant.

### Effects of experimental condition on perceptual and physiological responses during the time to exhaustion tests

**Fig. 4:**
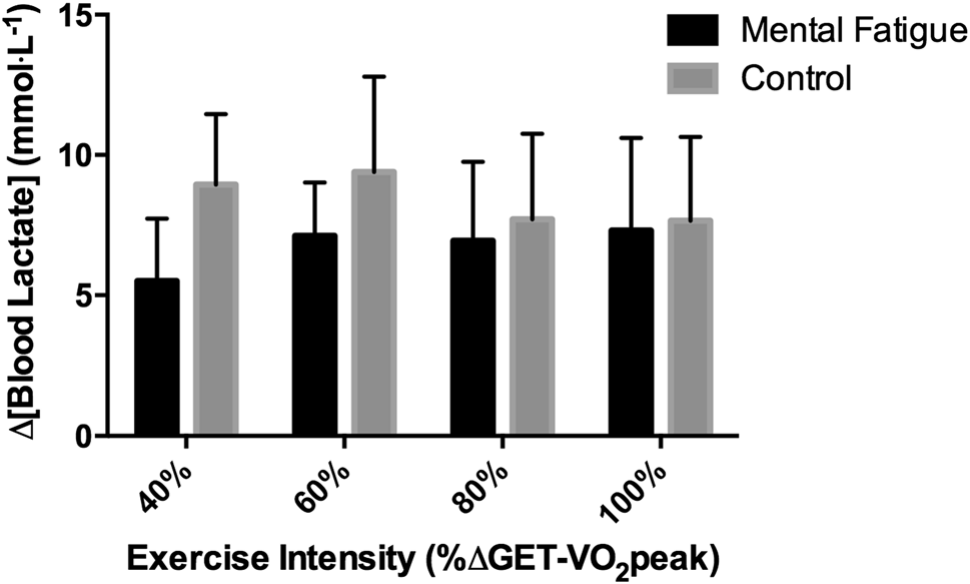
Blood lactate accumulation during the time to exhaustion tests used in the determination of CP and W′

There was no condition x intensity interaction effect for the pre–post change in blood lactate concentration measured from each TTE test (*P* > 0.05). However, a significant main effect was evident, with blood lactate accumulation being significantly lower in the MF compared to the CON condition (*P* < 0.05; Fig. 4). There was no significant effect of TTE intensity on blood lactate accumulation (*P* > 0.05).

**Fig. 5:**
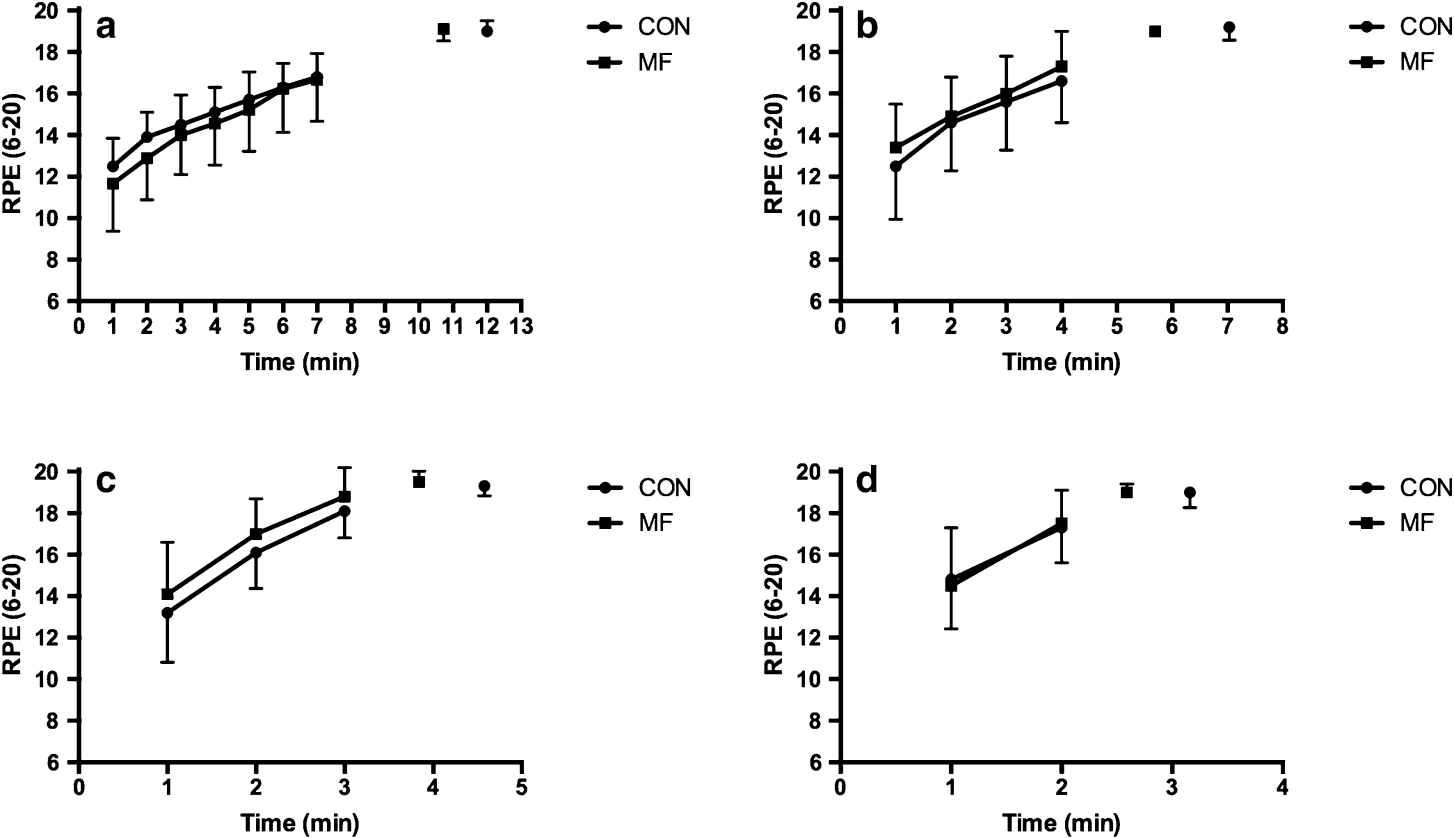
Rating of perceived exertion (RPE) during the time to exhaustion tests used in the determination of CP and W′ in the mental fatigue (MF) and control (CON) conditions: **a** 40%Δ, **b** 60%Δ, **c** 80%Δ and **d** 100% $\dot {V}{{\text{O}}_{{\text{2peak}}}}$

A significant interaction effect of condition x intensity was evident for the slope of the RPE response (*P* < 0.05). Figure 5 and Table 2 provides details of RPE and the slope of the RPE response at each exercise intensity in both conditions. The slope of the response was significantly greater in the MF vs. CON condition at exercise intensities equivalent to 40%Δ, 60%Δ and 100% $\dot {V}{{\text{O}}_{{\text{2peak}}}}$ (*P* < 0.05).

**Table 2:** Effects of experimental condition on the slope of the rating of perceived exertion (RPE) response at 40%Δ 60%Δ, 80%Δ and 100% for the mental fatigue (MF) and control (CON) conditions

	40%Δ	60%Δ	80%Δ	100% $\dot {V}{{\text{O}}_{{\text{2peak}}}}$
CON	0.61 ± 0.15	1.19 ± 0.35	1.78 ± 0.67	2.06 ± 0.92
MF	0.76 ± 0.21*	1.27 ± 0.34*	2.31 ± 0.97	3.31 ± 0.61*

Values are RPE slope (RPE min^−1^) presented as mean ± SD

*Significant different to CON condition (*P* < 0.05)

There was no interaction effect between condition x intensity for the slope of the HR response (*P* > 0.05). The slope of the HR response was not significantly different between MF and CON conditions (slope = 8 ± 3 vs. 7 ± 3 beats.min^−1^ for MF and CON conditions respectively; *P* > 0.05). The maximal HR (grand mean = 178 ± 3 vs. 179 ± 3 beats.min^−1^ for MF and CON respectively), and RPE (Fig. 5) recorded during each TTE were not different between conditions (*P* > 0.05).

## Discussion

To our knowledge, this is the first study to demonstrate that the W′ can be altered by purely psychological factors. Specifically, we used a prolonged and demanding cognitive task to induce mental fatigue, and thus reduce TTE during the high-intensity cycling exercise tests used to construct the work-time relationship. The shorter TTE did not alter the slope of the relationship, hence CP was not affected, but the intercept was lower following mental fatigue, resulting in a reduced W′.

The participant’s greater perception of mental fatigue following the Stroop task compared to reading fitness magazines (Fig. 2a) suggests that the MF condition was successful in inducing a higher level of mental fatigue, without changing perceived physical fatigue (Fig. 2b), compared to the CON condition. Supporting the findings of previous research (Marcora et al. 2009b; Pageaux et al. 2014), we found a significant impairment of endurance performance with mental fatigue in our group of trained cyclists. Specifically, TTE was reduced by an average of ~ 15% across exercise intensities equivalent to Δ40, Δ60, Δ80, and 100% $\dot {V}{{\text{O}}_{{\text{2peak}}}}$ (Table 2). These findings are also in agreement with the findings of Bray et al. (2008) who demonstrated that as little as 220 s of mental exertion involving response inhibition could reduce the endurance capacity of the muscles involved in a hand grip exercise. However, even though we found a reduced TTE during cycling exercise, this did not alter the slope of the work–time relationship, used to estimate CP (Fig. 3a). From a mathematical perspective, this is likely due to the reductions in TTE being proportional across the different exercise durations. From a physiological perspective, it is plausible to suggest that mental fatigue does not affect the purportedly ‘aerobic’ component of the work–time relationship (i.e. the CP). However, the reduced TTE did affect the W′ (intercept of the work–time relationship. Fig. 3b), due to participants disengaging from the TTE test sooner in the MF vs. CON condition. Previous research suggests that this disengagement was not due to mental fatigue altering the underlying physiological processes of central or peripheral muscle fatigue (Marcora et al. 2009b; Pageaux et al. 2014, 2015), rather the participants experiencing a higher than normal perception of effort in the MF condition (Fig. 5). Thus, the higher than normal perception of effort likely reduced the fixed amount of work that participants could perform above the CP before task disengagement occurred, regardless of the rate at which the work was done.

The level of participant motivation was not significantly different immediately prior to the TTE tests in both MF and CON conditions. Therefore, to understand the negative effect of the mental fatigue intervention on endurance performance, it is important to consider the perception of effort. In this regard, findings from previous research have demonstrated impairment in endurance performance without alternations in cardiorespiratory, metabolic and neuromuscular responses to endurance exercise (Marcora et al. 2009b; Pageaux et al. 2013, 2014), following mental fatigue. These studies suggest that following a period of prolonged mental exertion (such as the 30-min Stroop task), central alterations result in a higher perception of effort and reduced endurance performance. Indeed, results of the current study demonstrate that the slope of the RPE response increased significantly over the different TTE intensities in both conditions, but were greater following mental fatigue (Fig. 5a, b). Mentally fatigued participants experienced a quicker rise in the perception of effort, meaning they reached their maximal level of perceived exertion sooner, and disengaged from the TTE test earlier, than in the CON condition. In other words, as postulated by Brehm’s motivational intensity theory (Wright 2008), our highly motivated participants withdrew effort (i.e. disengaged) when the task was perceived to be too difficult. Therefore, the increased perception of effort and reduced TTE is likely to impact upon the mathematical work–time relationship used to estimate the CP and W′. Interestingly, Nakamura et al. (2005) propose that the perception of effort and CP are “indirectly” related, and that a “perception of effort threshold” (derived from linear extrapolation of the relationship between exercise intensity and perceived exertion rate), can be used to determine CP derived from a series of TTE tests. However, we did not find any change in CP using mental fatigue to experimentally manipulate the perception of effort. Instead participant early disengagement from the TTE test following mental fatigue meant that they were unlikely to have expended their entire W′, even though they exercise until volitional exhaustion. This hypothesis is supported by the blunted blood lactate accumulation seen during the TTE tests in the MF condition. As a consequence, this data suggests that models of endurance performance which purely focuses on physiological mechanisms (as in the CP concept), fail to encapsulate all of the determinants of performance during endurance performance. Moreover, because 30 min of mental exertion increased perception of effort and reduced TTE performance, sports scientist and coaches should instruct study participants or athletes to avoid undertaking prolonged cognitive tasks (e.g. academic study or working at a computer), prior to exercise testing for determination of the CP and W′. In addition, endurance athletes should avoid prolonged periods of mental exertion prior to training and competition when the aim is to perform optimally.

## Conclusions

The critical power concept is defined by the mathematical relationship between work done and TTE within the severe-intensity exercise domain, and represents a threshold above which the individual progressively expends the W′. The results from this study suggest that the parameter of W′ can be altered by psychological factors (i.e. mental fatigue). Specifically, the higher than normal perception of effort experienced in the MF condition reduced participants’ TTE as they reached their maximal perception of effort in a shorter period of time, in turn reducing W′, previously purely attributed to muscle physiology. The findings of our research therefore suggest that attempting to constrain the mathematical work–time relationship by purely physiological parameters seems problematic and overly restrictive.

## Abbreviations

CON: Control
CP: Critical power
GET: Gaseous exchange threshold
H^+^: Hydrogen ion
HR: Heart rate
MF: Mental fatigue
PCr: Phosphocreatine
PE: Perception of effort
Pi: Inorganic phosphate
Rev min^−1^: Revolutions per minute
RPE: Rating of perceived exertion
TTE: Time to exhaustion
VE: Ventilation
$$\dot {V}{{\text{O}}_{\text{2}}}$$: Oxygen consumption
$\dot {V}{{\text{O}}_{{\text{2peak}}}}$: Peak oxygen consumption
$\dot {V}{\text{C}}{{\text{O}}_{\text{2}}}$: Volume of carbon dioxide produced
W′: W prime
